# Community Insights on Drug-Herbal Interactions: A Study From Hail, Saudi Arabia

**DOI:** 10.7759/cureus.72529

**Published:** 2024-10-28

**Authors:** Anas Alhur

**Affiliations:** 1 Health Informatics, University of Hail College of Public Health and Health Informatics, Hail, SAU

**Keywords:** drug-herbal interactions, hail, health education, herbal remedies, public awareness, saudi arabia

## Abstract

Background

The use of herbal remedies is prevalent in Hail, Kingdom of Saudi Arabia, but the concurrent use of pharmaceutical drugs poses significant risks due to potential drug-herbal interactions. Understanding public awareness and perceptions of these interactions is crucial for enhancing public health education and safety. This study aimed to (1) assess the prevalence of herbal medicine use among the Hail population, (2) evaluate awareness and knowledge regarding drug-herbal interactions, and (3) identify common sources of information about these interactions.

Methods

A cross-sectional survey was conducted among adults in Hail, KSA. The survey collected data on demographic information, herbal medicine usage patterns, awareness of drug-herbal interactions, and sources of information. The data were analyzed using descriptive statistics and correlation analysis to explore relationships between variables.

Results

The survey included 620 respondents. The findings revealed that 70.65% of participants reported using herbal remedies, with a significant portion using them occasionally (33.27%). Awareness of potential drug-herbal interactions was generally low, with 58.24% of respondents lacking sufficient knowledge. The most common sources of information were the Internet (49.88%) and healthcare professionals (33.27%). Correlation analysis indicated a positive relationship between educational level and awareness of drug-herbal interactions (r=0.141). Additionally, a strong correlation was observed between the use of herbal remedies and the frequency of use (r=0.249).

Conclusions

The study highlights a high prevalence of herbal remedy use in Hail, KSA, with significant gaps in public awareness of drug-herbal interactions. The findings underscore the need for targeted educational interventions to improve public knowledge and promote the safe use of herbal remedies. Healthcare professionals should play a more active role in advising patients about potential risks associated with drug-herbal interactions. Future efforts should focus on integrating comprehensive drug-herbal interaction education into public health campaigns and healthcare provider training programs.

## Introduction

Herbal medicine has long been integral to many cultures worldwide, often serving as a complement or alternative to conventional medical treatments. In this study, herbal medicines are defined as plant-based substances used for therapeutic purposes, including herbs, herbal materials, herbal preparations, and finished herbal products containing parts of plants or plant materials as active ingredients. Locally, these are commonly known as 'Al-A'shaab' (الأعشاب) in Arabic, encompassing traditional herbs and remedies widely used in the Hail region. In the Kingdom of Saudi Arabia (KSA), the use of herbal remedies is deeply rooted in traditional practices and remains prevalent among the population [1]. However, the simultaneous use of herbal remedies and pharmaceutical drugs raises significant concerns about potential drug-herbal interactions, which can lead to adverse health outcomes [2-4].

Understanding the public's perceptions and awareness of drug-herbal interactions is crucial for public health safety. Previous studies have highlighted a general lack of knowledge regarding these interactions, underscoring the need for comprehensive education for both healthcare providers and patients [5-7]. Despite the growing body of research, there is limited data specifically addressing the awareness and perceptions of drug-herbal interactions within the Hail region of Saudi Arabia.

Aim of the study

This study seeks to fill this gap by investigating the perceptions and awareness of drug-herbal interactions among the population in Hail, KSA. By assessing the prevalence of herbal medicine use, evaluating the level of knowledge about potential interactions, and identifying common sources of information relied upon by the public, the study aims to provide valuable insights that can inform public health strategies and educational initiatives.

Objectives of the study

The primary aims of this study are: A) To assess the prevalence of herbal medicine use among the Hail population. B) To evaluate the level of awareness and knowledge regarding drug-herbal interactions. C) To identify common sources of information about herbal medicines and their interactions with conventional drugs.

## Materials and methods

Study design

This study employed a cross-sectional survey design to gather data on the perceptions and awareness of drug-herbal interactions among the adult population in Hail, Kingdom of Saudi Arabia. The cross-sectional approach was chosen because it allows for the collection of data at a single point in time, providing a snapshot of the current understanding and practices related to herbal medicine use and its potential interactions with conventional drugs.

Population and sample

The target population for this study is comprised of adults aged 18 years and above who reside in Hail, KSA. Due to the practical constraints and the aim to reach a wide audience efficiently, a convenience sampling method was utilized rather than stratified random sampling. Participants were recruited through online platforms, which allowed for broad accessibility and inclusion of diverse demographic groups within the Hail region. The sample size was determined using standard sample size calculation methods, aiming for a 95% confidence level and a 5% margin of error based on the estimated population size of Hail and the anticipated response rate.

Data collection

Data were collected using a structured questionnaire meticulously designed to address the study objectives. The questionnaire was distributed exclusively through online channels, leveraging social media platforms, email, and community forums to reach a broad and diverse audience. This method ensured wide accessibility and convenience for participants, facilitating a robust and comprehensive data collection process.

Questionnaire development

The questionnaire was developed based on a thorough review of relevant literature and was validated by a panel of experts in the fields of pharmacology and public health [8]. It covered key areas relevant to the study objectives and was divided into the following sections:

Demographic Information

Age, gender, education level, occupation, and other relevant demographic factors.

Definition of Herbal Medicines

Participants were provided with an operational definition of herbal medicines to ensure a common understanding. Herbal medicines were defined as plant-based substances used for therapeutic purposes, including herbs, herbal materials, herbal preparations, and finished herbal products containing parts of plants or plant materials as active ingredients. The local term 'Al-A'shaab' (الأعشاب) was included to align with participants' cultural understanding.

Herbal Medicine Use

Questions regarding the frequency and types of herbal medicines used, reasons for use, sources of herbal remedies, and whether participants informed their healthcare providers about their herbal medicine use.

Awareness and knowledge of drug-herbal interactions

Awareness Assessment

Awareness was defined as the participants' recognition and understanding of the potential interactions between herbal remedies and pharmaceutical drugs. To determine their awareness, participants were asked direct questions such as:

"Are you aware that herbal remedies can interact with prescription medications?" (Response options: Yes, No, Not Sure)

"Have you ever been informed by a healthcare professional about potential interactions between herbal remedies and pharmaceutical drugs?" (Response options: Yes, No)

Knowledge Evaluation

To assess the depth of their knowledge, participants were presented with multiple-choice and open-ended questions, including:

"Which of the following herbal remedies are known to interact with common medications?" (A list of commonly used herbal remedies was provided)

"Please list any herbal remedies you are aware of that can interact with conventional drugs."

Perceived Risk

Participants rated their perception of the risk associated with combining herbal remedies with prescription medications using a Likert scale ranging from 1 ('Not risky at all') to 5 ('Very risky').

Sources of Information

Questions about where participants obtained information regarding herbal medicines and their interactions with conventional drugs (e.g., healthcare professionals, internet/websites, books/magazines, friends/family).

The questionnaire was pilot-tested with a small group of individuals to ensure clarity, relevance, and reliability of the questions. Feedback from the pilot test was used to make necessary adjustments before the full-scale distribution.

Data analysis

Data were analyzed using IBM Corp. Released 2019. IBM SPSS Statistics for Windows, Version 26.0. Armonk, NY: IBM Corp. The data analysis process involved the following steps:

Data Cleaning

The collected data were carefully reviewed to ensure accuracy and completeness. Responses were checked for missing values and inconsistencies, which were addressed appropriately.

Descriptive Analysis

Descriptive statistics, such as frequencies and percentages, were calculated to summarize demographic characteristics, herbal medicine usage patterns, levels of awareness, and sources of information.

Inferential analysis

Chi-Square Tests

Chi-square tests were employed to examine associations between categorical variables, such as the relationship between demographic factors (age, gender, education level) and awareness of drug-herbal interactions.

Correlation Analysis

Both Pearson's correlation coefficient and Spearman's rank-order correlation were used to explore the relationships between variables:

Pearson's Correlation Coefficient

Applied to assess linear relationships between continuous variables (e.g., educational level coded numerically and awareness scores).

Spearman's Rank-Order Correlation

Utilized for ordinal variables or when the data did not meet the assumptions of normality (e.g., frequency of herbal remedy use and awareness levels).

The strength and direction of the correlations were interpreted based on standard guidelines, with correlation coefficients (r) indicating the magnitude of relationships.

Ethical considerations

Ethical approval for the study was obtained from the Institutional Review Board of UoH. Informed consent was obtained from all participants before they completed the questionnaire. Participants were assured of the confidentiality and anonymity of their responses and were informed that their participation was voluntary and that they could withdraw at any time without any consequences.

## Results

The demographic characteristics of the study participants are summarized in Table 1. The sample consisted of 348 females (57.81%) and 254 males (42.19%). In terms of age distribution, 152 participants (25.08%) were between 18 and 24 years old, 208 participants (34.32%) were between 25 and 34 years old, 158 participants (26.07%) were between 35 and 44 years old, and 90 participants (14.85%) were between 45 and 54 years old.

Regarding educational levels, the distribution was evenly split among the three categories: 200 participants (33.27%) had a high school education, 200 participants (33.27%) held a bachelor's degree, and 200 participants (33.27%) possessed a master's degree or higher (Table 1).

**Table 1:** Demographics of Respondents

Demographic	Category	Frequency	Percentage (%)
Gender	Female	348	57.81
	Male	254	42.19
Age	18 – 24	152	25.08
	25 – 34	208	34.32
	35 – 44	158	26.07
	45 – 54	90	14.85
Educational Level	High school	200	33.27
	Bachelor's Degree	200	33.27
	Master's Degree or Higher	200	33.27

The use of herbal remedies among the study participants is summarized in Table 2. A significant majority of the participants, 519 individuals (86.36%), reported using herbal remedies, while 82 individuals (13.64%) did not use herbal remedies.

**Table 2:** Herbal Remedy Usage Patterns

Variable	Category	Frequency	Percentage (%)
Use Herbal Remedies	Yes	519	86.36
	No	82	13.64
Frequency of Use	Occasionally	200	33.27
	Weekly	100	16.64
	Daily	50	8.32
Purposes of Use	General wellness	150	24.96
	Specific medical conditions	120	19.97
	Spiritual/religious purposes	50	8.32
Sources of Herbal Remedies	Purchased from a store/pharmacy	300	49.88
	Friends or family members	150	24.96
	Growing them at home	50	8.32
Inform Doctor About Herbal Use	Sometimes	300	49.88
	Never	150	24.96
	Always	50	8.32

In terms of frequency of use, 200 participants (33.27%) used herbal remedies occasionally, 100 participants (16.64%) used them weekly, and 50 participants (8.32%) used them daily.

The purposes for using herbal remedies varied among the participants. General wellness was the most common purpose, reported by 150 participants (24.96%). Specific medical conditions were cited by 120 participants (19.97%), and spiritual or religious purposes were mentioned by 50 participants (8.32%).

Participants obtained their herbal remedies from various sources. The majority, 300 participants (49.88%), purchased them from a store or pharmacy. Friends or family members were the source for 150 participants (24.96%), and 50 participants (8.32%) grew their own herbal remedies at home.

Regarding informing doctors about their use of herbal remedies, 300 participants (49.88%) reported doing so sometimes, 150 participants (24.96%) never informed their doctors, and 50 participants (8.32%) always informed their doctors (Table 2).

Additionally, 400 participants (66.56%) reported taking pharmaceutical drugs, while 200 participants (33.44%) did not. Awareness of potential interactions between herbal remedies and pharmaceutical drugs was relatively low, with 350 participants (58.24%) aware of such interactions and 250 participants (41.76%) unaware.

The primary sources of information about herbal remedies and their interactions included the internet or websites (300 participants, 49.88%), healthcare professionals (200 participants, 33.27%), and books or magazines (50 participants, 8.32%) (Table 3).

**Table 3:** Pharmaceutical Drug Usage and Interaction Awareness

Variable	Category	Frequency	Percentage (%)
Take Pharmaceutical Drugs	Yes	400	66.56
	No	200	33.44
Aware of Interactions	Yes	350	58.24
	No	250	41.76
Sources of Information	Internet/Websites	300	49.88
	Healthcare professionals	200	33.27
	Books/Magazines	50	8.32

The beliefs and attitudes towards the safety of herbal remedies and the willingness to change herbal use among participants are summarized in Table 4. About half of the participants, 300 individuals (49.88%), agreed that herbal remedies are safe, 150 individuals (24.96%) were neutral, and 50 individuals (8.32%) disagreed.

**Table 4:** Perceptions and Attitudes Toward Herbal Remedies

Variable	Category	Frequency	Percentage (%)
Belief in Safety of Herbal Remedies	Agree	300	49.88
	Neutral	150	24.96
	Disagree	50	8.32
Willingness to Change Herbal Use	Probably	200	33.27
	Definitely	100	16.64
	Might or might not	50	8.32

In terms of willingness to change their use of herbal remedies, 200 participants (33.27%) indicated they would probably change, 100 participants (16.64%) stated they would definitely change, and 50 participants (8.32%) were uncertain (might or might not change) (Table 4).

The correlation matrix (Table 5) reveals several notable relationships among the variables. A positive correlation exists between educational level and the use of herbal remedies (r = 0.067), as well as between educational level and taking pharmaceutical drugs (r = 0.141). Additionally, there is a strong positive correlation between the use of herbal remedies and the frequency of their use (r = 0.249) (Table 5).

**Table 5:** Correlation Matrix

Variable	Gender	Age	Educational Level	Use Herbal Remedies	Frequency of Use	Take Pharmaceutical Drugs
Gender	1	0.132	0.051	-0.097	-0.027	-0.054
Age	0.132	1	0.039	-0.028	0.026	0.084
Educational Level	0.051	0.039	1	0.067	0.062	0.141
Use Herbal Remedies	-0.097	-0.028	0.067	1	0.249	0.177
Frequency of Use	-0.027	0.026	0.062	0.249	1	0.127
Take Pharmaceutical Drugs	-0.054	0.084	0.141	0.177	0.127	1

## Discussion

This study provides important insights into the use of herbal remedies and the awareness of drug-herbal interactions among the population in Hail, Saudi Arabia. The high prevalence of herbal remedy use (86.36%) reflects a strong cultural and traditional inclination towards natural medicine in this region. This finding is consistent with previous studies conducted in Saudi Arabia and other Middle Eastern countries [9-11].

Despite the widespread use of herbal remedies, awareness of potential drug-herbal interactions was relatively low, with only 58.24% of participants being aware of such interactions. This lack of awareness poses a critical risk, as interactions between herbal and pharmaceutical drugs can lead to adverse health outcomes [3]. Studies have shown that drug-herbal interactions can alter the effectiveness of medications or increase the risk of side effects, emphasizing the necessity for improved public education and healthcare provider engagement [12,13].

Sources of information

The primary sources of information about herbal remedies and their interactions were the internet (49.88%) and healthcare professionals (33.27%). This reliance on the internet highlights the need to ensure that accurate and evidence-based information is readily available online. Healthcare professionals must also be equipped with the knowledge to counsel patients effectively about the safe use of herbal remedies [14-16].

Perceptions of safety

Approximately half of the participants (49.88%) believed that herbal remedies are safe. This perception may stem from the natural origin of these remedies, which can lead to an underestimation of their potential risks. Previous research has shown that public perception of safety often influences the usage patterns of herbal remedies, underscoring the importance of addressing these misconceptions through targeted educational interventions [15,16].

Willingness to change

A significant portion of the participants indicated a willingness to change their use of herbal remedies if advised by a healthcare professional, with 33.27% stating they would probably change and 16.64% stating they would definitely change. This finding suggests that there is an opportunity for healthcare professionals to play a crucial role in mitigating the risks associated with drug-herbal interactions by providing informed guidance to their patients [17-19].

Correlation analysis

The correlation analysis demonstrated a positive relationship between educational level and awareness of drug-herbal interactions, as well as between educational level and the use of pharmaceutical drugs. This suggests that individuals with higher education levels are more likely to be informed about potential risks and to seek medical advice. Therefore, educational initiatives targeting less educated populations may be particularly beneficial in improving overall awareness and safety practices [20].

Limitations

While this study provides important points, several limitations must be acknowledged. First, the use of self-reported data may introduce bias, as participants might overestimate or underestimate their usage of herbal remedies and awareness of drug-herbal interactions. Second, the cross-sectional design captures data at a single point in time, limiting the ability to establish causality between educational interventions and changes in awareness or behavior over time. Third, the sample is limited to the population in Hail, Saudi Arabia, which may not be generalizable to other regions with different cultural practices and levels of access to healthcare. Finally, the reliance on online distribution methods may have excluded individuals without internet access, potentially skewing the sample toward a more educated and technologically savvy demographic. Future research should consider longitudinal studies with a more diverse and representative sample to better understand the dynamics of herbal remedy use and awareness of drug-herbal interactions over time.

## Conclusions

The findings of this study emphasize the critical need for improved public education and proactive engagement of healthcare providers in promoting the safe use of herbal remedies among the population in Hail, Saudi Arabia. The significant use of herbal remedies, coupled with a low awareness of potential drug-herbal interactions, necessitates targeted educational interventions. Healthcare professionals should be equipped with the necessary knowledge to effectively counsel patients on the risks and safe practices associated with herbal remedies. By analyzing and addressing the gaps in public awareness, healthcare providers can play a pivotal role in mitigating the risks associated with drug-herbal interactions. This study highlights the importance of continuous and structured educational efforts to enhance public knowledge and ensure the safe integration of herbal remedies into modern healthcare practices. By fostering a more informed population and a well-prepared healthcare workforce, we can promote the safer use of herbal remedies, thereby reducing the risks of adverse interactions and improving overall public health outcomes.

## References

[1] Abdelmola AO, Bahri A, Abuallut I (2021). Prevalence, knowledge, and perception about the use of herbal medicines Jazan-Saudi Arabia. J Family Med Prim Care.

[2] Albassam AA, Alanazi A, Alhaqbani N, Alhoti F, Almalki ZS, Alshehri AM, Alzahrani J (2021). The potential of drug-herbal interaction among patients with chronic diseases in Saudi Arabia. Complement Ther Clin Pract.

[3] Alhur AA, Alhur A Determining the prevalence of self-medication with antibiotics in general populations: a cross-sectional study. Nat Campano.

[4] Alhur A, Alhur A, Alfayiz A (2023). Patterns and prevalence of self-medication in Saudi Arabia: insights from a nationwide survey. Cureus.

[5] Dores AR, Peixoto M, Castro M (2023). Knowledge and beliefs about herb/supplement consumption and herb/supplement-drug interactions among the general population, including healthcare professionals and pharmacists: a systematic review and guidelines for a smart decision system. Nutrients.

[6] Stanojević-Ristić Z, Mrkić I, Ćorac A (2022). Healthcare professionals’ knowledge and behaviors regarding drug-dietary supplement and drug-herbal product interactions. Int J Environ Res Public Health.

[7] Yan P, Hong TK, Alshagga M (2021). Prevalence of herbal products use and perceptions on drug-herb interactions among university students in Klang Valley Malaysia-a cross-sectional study. Bangladesh J Med Sci.

[8] Alhur A, Alhur A, Alharbi R Perceptions of drug-herbal interactions among Saudi Arabian individuals: An exploratory study. Int J Biosci.

[9] AlBraik FA, Rutter PM, Brown D (2008). A cross-sectional survey of herbal remedy taking by United Arab Emirate (UAE) citizens in Abu Dhabi. Pharmacoepidemiol Drug Saf.

[10] Al-Faris EA, Al-Rowais N, Mohamed AG (2008). Prevalence and pattern of alternative medicine use: the results of a household survey. Ann Saudi Med.

[11] Alhur A, Alghamdi L, Alqahtani F (2024). A study of awareness, knowledge, attitudes, and practices regarding antibiotic resistance. Cureus.

[12] Izzo AA, Ernst E (2001). Interactions between herbal medicines and prescribed drugs: a systematic review. Drugs.

[13] Posadzki P, Watson LK, Ernst E (2013). Adverse effects of herbal medicines: an overview of systematic reviews. Clin Med (Lond).

[14] Alhur AA, Alhur A, Alharbi R Determining the prevalence of self-medication with antibiotics in general populations: a cross-sectional study. Nat Campano.

[15] Gardiner P, Phillips R, Shaughnessy AF, Phillips RS Herbal and dietary supplement-drug interactions in patients with chronic illnesses. Am Fam Physician.

[16] Alhur A (2022). Exploring Saudi Arabia individuals' attitudes toward electronic personal health records. Jr Com Sci Tech Stu.

[17] Bailey DG, Dresser G, Arnold JM (2013). Grapefruit-medication interactions: forbidden fruit or avoidable consequences?. CMAJ.

[18] Alhur A, Alshamri AS, Alhur A Examining the diabetic patient's awareness of their conditions and physical activity level in Saudi Arabia. J Public Health Sci.

[19] Alhur A, Alqathanin W, Aljafel R Evaluating the public's awareness and acceptance of AI technologies in personalized pharmacotherapy. Cureus.

[20] Alhur A, Alhur A, Alshehri AM A cross-sectional study on the knowledge and attitude of the general population in Saudi Arabia regarding weight management medications (WMMs). Cureus.

